# Identification of copy number variants from exome sequence data

**DOI:** 10.1186/1471-2164-15-661

**Published:** 2014-08-07

**Authors:** Pubudu Saneth Samarakoon, Hanne Sørmo Sorte, Bjørn Evert Kristiansen, Tove Skodje, Ying Sheng, Geir E Tjønnfjord, Barbro Stadheim, Asbjørg Stray-Pedersen, Olaug Kristin Rødningen, Robert Lyle

**Affiliations:** Department of Medical Genetics, University of Oslo, Oslo, Norway; Department of Medical Genetics, Oslo University Hospital, Postboks 4956, Oslo, 0424 Norway; Department of Haematology, Oslo University Hospital, Oslo, Norway; Institute of Clinical Medicine, University of Oslo, Oslo, Norway; Center for Human Immunobiology/Section of Immunology, Allergy, and Rheumatology, Texas Children’s Hospital, Houston, TX USA; Baylor-Hopkins Center for Mendelian Genomics of the Department of Molecular and Human Genetics, Baylor College of Medicine, Houston, TX USA

**Keywords:** Exome, CNV prediction, Custom aCGH

## Abstract

**Background:**

With advances in next generation sequencing technologies and genomic capture techniques, exome sequencing has become a cost-effective approach for mutation detection in genetic diseases. However, computational prediction of copy number variants (CNVs) from exome sequence data is a challenging task. Whilst numerous programs are available, they have different sensitivities, and have low sensitivity to detect smaller CNVs (1–4 exons). Additionally, exonic CNV discovery using standard aCGH has limitations due to the low probe density over exonic regions. The goal of our study was to develop a protocol to detect exonic CNVs (including shorter CNVs that cover 1–4 exons), combining computational prediction algorithms and a high-resolution custom CGH array.

**Results:**

We used six published CNV prediction programs (ExomeCNV, CONTRA, ExomeCopy, ExomeDepth, CoNIFER, XHMM) and an in-house modification to ExomeCopy and ExomeDepth (ExCopyDepth) for computational CNV prediction on 30 exomes from the 1000 genomes project and 9 exomes from primary immunodeficiency patients. CNV predictions were tested using a custom CGH array designed to capture all exons (exaCGH). After this validation, we next evaluated the computational prediction of shorter CNVs. ExomeCopy and the in-house modified algorithm, ExCopyDepth, showed the highest capability in detecting shorter CNVs. Finally, the performance of each computational program was assessed by calculating the sensitivity and false positive rate.

**Conclusions:**

In this paper, we assessed the ability of 6 computational programs to predict CNVs, focussing on short (1–4 exon) CNVs. We also tested these predictions using a custom array targeting exons. Based on these results, we propose a protocol to identify and confirm shorter exonic CNVs combining computational prediction algorithms and custom aCGH experiments.

**Electronic supplementary material:**

The online version of this article (doi:10.1186/1471-2164-15-661) contains supplementary material, which is available to authorized users.

## Background

With advances in next generation sequencing technologies and genomic capture techniques, exome sequencing has become a cost-effective approach for mutation detection in genetic diseases [1]. The availability of efficient and robust analysis tools such as Genome Analysis Toolkit (GATK) makes it possible to discover SNPs and indels using sequence data with high sensitivity and specificity [2]. Even though CNV Copy Number Variant discovery in whole-genome sequence data is performed with high accuracy [3], a number of different algorithms with variable specificities are available to detect CNVs in exome data [4]. Thus, selecting the correct algorithm or algorithm combination has become a bottleneck of exome CNV prediction. Moreover, CNV detection algorithms have low specificity and sensitivity in predicting small CNVs (1 to 4 exons) [5].

While computational approaches have limitations in predicting CNVs in exome sequence data, standard array comparative genomic hybridization (aCGH) used for genome-wide high-resolution CNV detection also show restrictions in exonic CNV detection due to the low probe coverage over exonic regions. Thus, the detection of exonic CNVs using both computational and aCGH based methods remains a challenging task.

The goal of our study was to develop a protocol to detect exonic CNVs (including CNVs that cover 1 to 4 exons) from exome sequencing data by combining computational prediction algorithms and a high-resolution custom CGH array. In this study, we predicted CNVs in 30 exomes obtained from the 1000 genomes project [6] using six recently published CNV detection programs along with an in-house modified algorithm. Computational CNV predictions were then tested using a custom CGH array focused on exonic regions. Next, true CNVs were identified by comparing computational predictions with the results of the CGH array. With the experimental validation of the computational predictions, the sensitivity and false positive rate of each program, or program combination, was determined. Results of the computational prediction demonstrated a wide range in both CNV count and size. Finally, we studied the clinical utility of the algorithms used in our study by computational prediction and array confirmation of CNVs in 9 exomes from primary immunodeficiency patients.

## Methods

### Data sets

Alignment data (BAM files) required for computational CNV prediction were obtained from the public data repository of the 1000 genomes exome project which targets the CCDS gene set in 2500 individuals [7]. All of the individuals are from the CEU population. DNA samples of the same exomes used in computational CNV predictions were obtained from the Coriell Institute for Medical Research [8].

### Exome sequencing

Exome capture was performed on primary immunodeficiency patient DNA using the Agilent SureSelect Human All Exome capture kit v.5 (Agilent Technologies), and captured libraries were sequenced on an Illumina HiSeq 2000. Sequence alignment was performed with Novolign (V2.07.17) [9]. Next, using GATK (V2.4-9) [2], the initial BAM files were realigned and the base quality scores were recalibrated. After marking the duplicates with Picard (V1.74) [10], the final set of alignment data (BAM files) required for computational CNV prediction were generated.

### Computational CNV prediction

The CNV analysis in our study has two main sections: computational CNV prediction and validation using a custom CGH array (Additional file 1: Figure S1). Computational CNV prediction was performed employing 6 CNV prediction programs (Table 1) and a complete listing of the parameters used for all the programs is presented in Additional file 1: Table S1.

**Table 1:** **Exome CNV prediction programs used in the study**

Program	Reference
ExomeCNV	https://secure.genome.ucla.edu/index.php/ExomeCNV_User_Guide [11]
CONTRA	http://sourceforge.net/apps/mediawiki/contra-cnv/index.php?title=Main_Page [12]
ExomeCopy	http://www.bioconductor.org/packages/2.9/bioc/html/exomeCopy.html [13]
ExomeDepth	http://cran.r-project.org/web/packages/ExomeDepth/index.html [14]
CoNIFER	http://conifer.sourceforge.net/index.html [5]
XHMM	http://atgu.mgh.harvard.edu/xhmm/index.shtml [15]

These programs use different statistical models in CNV calling. However, ExomeCopy and ExomeDepth use a similar statistical approach based on Hidden Markov Models. The main differences between these two programs are the implementation of exon length normalization (SubdivideGRange method) in ExomeCopy [13] and generation of aggregate reference set (select.reference.set method) in ExomeDepth [14]. As further discussed in results and discussion, these two programs exhibited the lowest and highest stringency in CNV calling. Therefore, to reach improved stringency in CNV calling and to utilize methods implemented in both ExomeCopy and ExomeDepth, we designed an algorithm (ExCopyDepth) combining these two programs (Flow and implementation of ExCopyDepth; Additional file 1: Figure S2). In ExCopyDepth, ExomeCopy is used for exon length normalization (SubdivideGRange method in ExomeCopy) and to extract read count and GC% data for these normalized exonic regions (CountBamInGRanges, GetGCcontent methods in ExomeCopy). Next, the most suitable reference dataset for each target exome was estimated using read count data from multiple samples (select.reference.set method in ExomeDepth). Finally, CNV regions were predicted using ExomeDepth (CallCNVs method). The initial tile length normalization performed in our method optimizes the generation of the aggregate reference set and minimizes the effect of GC content while CNV calling.

### Custom array design (exaCGH)

To verify the ability to computationally call exonic CNVs, a custom CGH array targeting exonic regions (exaCGH) was designed using the Agilent eArray web portal [16]. The design process was initiated by the Agilent High-Definition Probe (HD-Probe) search using a genomic interval list that specifies the 1000 genome exonic regions. As low probe scores are observed for GC-rich regions, it is difficult to design high-scoring probes for all exonic regions. In addition, Agilent guidelines recommend 150-200 bp minimum probe spacing for optimum probe hybridization. Therefore, in order to search high scoring probes and to reach the recommended spacing between probes, genomic intervals were designed by adding 800 bp flanking regions to either side of the exonic regions defined in the 1000 genomes exome project. Although the incorporation of 800 bp flanking regions extends the probe search into intronic regions, this shows no negative effect on detecting short CNVs since the probes are internal to the CNV. A flowchart of the exaCGH array design (data preparation, Agilent eArray HD-Search and evaluation of the search results) is presented in Additional file 1: Figure S3. The complete design, and capture regions of exaCGH, are available on request from the authors. Since probes covering the 1000 genomes exome do not occupy the whole 1×1M array, the remaining features were filled by probes covering exonic, CDS (coding regions) and CCDS (conserved coding) regions specified in the GENCODE v15 annotation [17]. Similar to the exome definition file used previously, 800 bp flanking regions were added to the 5′ and 3′ ends of the GENCODE v.15 regions (Additional file 1: Figure S3). After searching probes for the exon array, the microarray was generated according to eArray guidelines.

We then compared exaCGH with two commercially available platforms used to detect CNVs: Agilent 1×1M array and Affymetrix CytoScan HD. In order to compare these designs, we utilized exonic and CDS regions specified in GENCODE V.15 and 800 bp flanking regions were added to the 5′ and 3′ end. Next, we evaluated the probe count distribution of each design (Additional file 1: Figure S4). Probe distributions clearly show that only ~10% of probes of Affymetrix CytoScan HD and ~1% of probes of Agilent 1x1M mapped to GENCODE V.15 with at least 4 probes per exonic and CDS region (including 1600 bp flanking regions). However, in the exaCGH design, considering only regions containing at least 4 probes, 83.9% of probes map to GENCODE v.15. The analysis of probe distribution demonstrated that exonic coverage (with at least 4 probes) of exaCGH is over 99.28 MB of GENCODEv.15, which is significantly higher than the Agilent 1×1M (3.5 MB) and Affymetrix CytoScan HD (6.67 MB). Moreover, to reduce the amount of noise in CNV detection, it is recommended to only call CNVs with at least 4 probes when analyzing aCGH results. Thus, the exaCGH array is a much improved design for detecting exonic CNVs due to the high probe density over exonic regions and the high exonic coverage.

Next, exaCGH experiments were performed using 9 DNA samples from the 1000 genomes exome project and 9 DNA samples from primary immunodeficiency patients following Agilent protocol V. 6.3. Agilent Genomic Workbench was used to call CNV regions which were detected by at least by four probes (with minimum average absolute log ratio for deletion and duplication > =0.25).

## Results and discussion

### Computational CNV prediction using published algorithms

**Figure 1:**
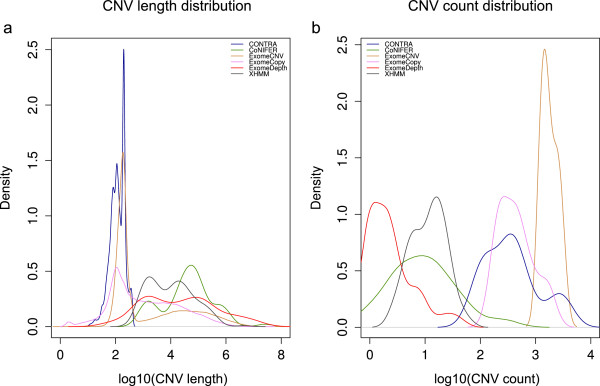
**Count and length distributions of CNVs predicted by the programs in the study. (a)** Length distribution of CNVs predicted by each program. **(b)** Count (number of CNVs) distribution of CNVs predicted by each program.

For computational CNV prediction we used six different programs and an algorithm (ExCopyDepth) combining ExomeCopy and ExomeDepth to detect CNVs from 30 exomes. Since these prediction algorithms depend on a reference dataset, it is essential that exomes are captured and sequenced using the same technology. However, since the technologies used to generate the 30 exomes obtained from the 1000 genomes project can be grouped into three different categories (Additional file 1: Table S2), all the exomes were categorized accordingly and separate computational CNV predictions were performed for each group.Results of the computational algorithms show striking variation in the length and number of CNVs predicted by the different programs (Figure 1a, b). For example, CoNIFER has a count range of 1–248 and ExomeCopy has a count range of 158–1837. In order to directly compare programs at this level, we identified individual exons covered by CNVs (exCNVs) from each program.

**Figure 2:**
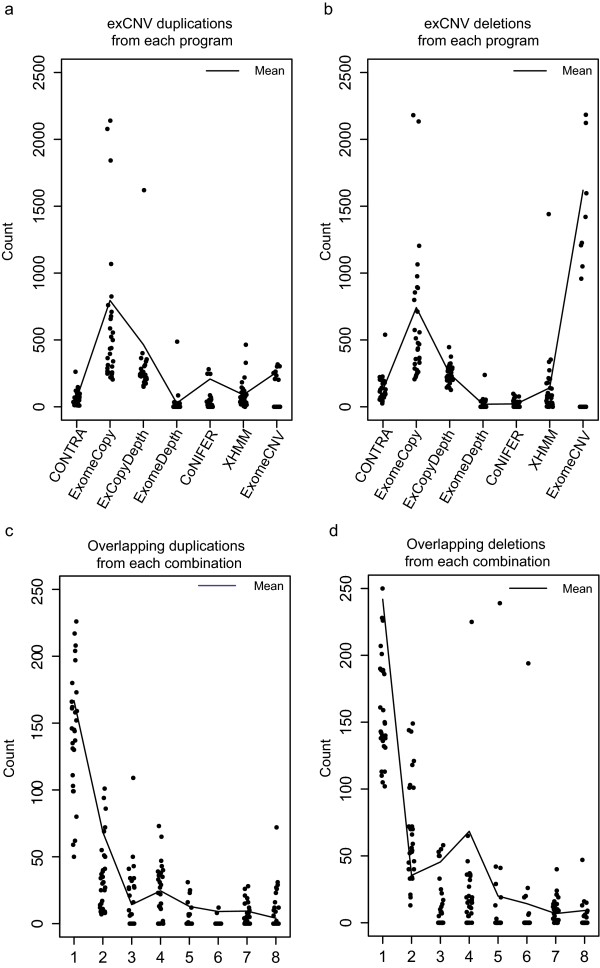
**exCNVs predicted by programs and overlapping exCNVs predicted by program combinations.** Each dot represents an individual exome. **(a)** Number of exonic duplications predicted by each program. **(b)** Number of exonic deletions predicted by each program. **(c)** Number of overlapping duplications predicted by each program combination. **(d)** Number of overlapping deletions predicted by each program combination. Program combinations **(c and d)**: 1, ExomeCopy/ExCopyDepth; 2, ExomeCopy/ExCopyDepth/CONTRA; 3, ExomeCopy/ExCopyDepth/CoNIFER; 4, ExomeCopy/ExCopyDepth/XHMM; 5, ExomeCopy/ExCopyDepth/ExomeDepth; 6, ExomeCopy/ExCopyDepth/ExomeDepth/XHMM; 7, ExomeCopy/ExCopyDepth/CONTRA/XHMM; 8, ExomeCopy/ExCopyDepth/CoNIFER/XHMM.

Analysis of exCNVs shows that the number of exCNVs predicted by computational programs differ considerably from each other (Figure 2a, b). For example, ExomeCopy predicted the highest mean number of exCNVs (1537) including 741 deleted and 795 duplicated exons. The lowest number of exCNVs was reported by ExomeDepth (average exonic CNV count 47, with 19 average number of deletions and 27 duplications). There are also large differences in the range of the number of exCNVs predicted by each program (Figure 2a, b). When comparing reference datasets used in the programs (Additional file 1: Table S1), ExomeCNV is the only program that uses a single sample (an external exome, NA12282) as a reference. This makes ExomeCNV results heavily dependent on depth of coverage distribution of the reference exome used. Thus ExomeCNV results were not utilized for downstream analysis.

### ExCopyDepth

As shown in previous studies, ExomeCopy demonstrated higher sensitivity compared to normalization and fast segmentation methods [18] in finding CNVs that overlap few exons [13]. A higher density over the 10 bp-10 kb range of CNV length distribution (Figure 1a) and the highest exCNV count compared to other programs (Figure 2a, b), may indicate the better performance of ExomeCopy in detecting shorter CNVs with the lowest stringency (which was tested and confirmed in the following stages of our study). Additionally, ExomeDepth has been optimized for detecting rare variants [14]. The lowest exCNV count observed for ExomeDepth compared to other programs (Figure 2a, b) may indicate the capability of ExomeDepth in detecting rare variants with higher stringency.

Since we are interested in detecting shorter CNVs with improved stringency, we combined different methods implemented in each of these programs in a modified algorithm, aiming to reach higher stringency compared to ExomeCopy and lower stringency compared to ExomeDepth. We reasoned that the higher stringency (lower CNV count) compared to ExomeCopy would have lower false positive CNV count compared to ExomeCopy, which was tested and confirmed in a later stage of our study.Evaluation of ExCopyDepth demonstrated an average of 711 exCNVs per exome, while other programs showed an average exCNV counts between 47 and 1537 (Figure 2a, b). The ExCopyDepth count (711) that falls away from the extreme ends of the exCNV count range (47–1537) exhibits the moderate stringency in CNV calling of ExCopyDepth.

### Identification of overlapping exCNVs

exCNV counts (from 1000 genome exomes) were spread over a wide range (0–2500; Figure 2a, b). We reasoned that intersecting (overlapping) exCNVs predicted by multiple programs would have a narrower range, and thus be a better estimate of true CNVs, which appeared to be the case (Figure 2c, d).The maximum average number of overlapping deletions and duplications (per program combination in Figure 2c, d) were 242 and 228 (from the intersection of ExomeCopy and ExCopyDepth), which were significantly lower than the highest average numbers resulting from individual programs (741 deletions and 795 duplications, Figure 2a, b). The number of overlapping exCNVs resulting from the intersection of three or more programs (combination 2 to 6 in Figure 2c, d) demonstrated a further decrease in exCNV count. The lower overlapping CNV counts from the intersection of ExomeCopy and ExCopyDepth compared to individual programs may imply lower false positive rates. We thus evaluated these predictions on a custom exon array.

### Validation of computational predictions using a custom exon array (exaCGH)

Since standard aCGH platforms exhibit low exonic coverage (Additional file 1: Figure S4), we designed a custom array (exaCGH) with at least 4 probes per exon (see Methods). Agilent aCGH microarrays have previously been reported to have the highest sensitivity in detecting single-copy alterations between 1 and 49 kb [19]. We ran nine 1000 genomes samples (Additional file 1: Table S3) with exaCGH to validate CNV predictions from each program. Additionally, since lower exCNV counts were shown by the intersection of ExomeCopy and ExCopyDepth (Figure 2c, d), these results were also used for experimental validation.

For experimental validation, true positives (TPs) were defined as CNVs which were detected by computational methods and confirmed by exaCGH. Next, the average true positive CNV count (AvgTP; for nine 1000 genomes samples) per program was calculated (Table 2). ExomeCopy and ExCopyDepth have a higher AvgTP compared to the other algorithms (Table 2). When considering the low AvgTP we observed for CoNIFER, ExomeDepth and XHMM, it is important to note that these programs are optimized for the identification of rare variants in large exomes data sets. Therefore, the use of a relatively small sample collection may contribute to the reported low TP CNV count.

**Table 2:** **True positive (TP)/false positive (FP) CNV ratio predicted from each program**

Program	AvgTP	TP/FP ratio	Total CNV count ^3^	Average CNVs per sample ^4^
CoNIFER	1.33 (1.33)^1^	0.92 (1.09)^2^	23	2.56
ExCopyDepth	28.11 (15.86)^1^	0.34 (0.51)^2^	422	46.89
ExomeCopy	289.33 (226.0)^1^	0.20 (0.21)^2^	11978	1330.89
ExomeDepth	1.11 (0.78)^1^	0.67 (0.70)^2^	17	1.89
Intersection of ExomeCopy / ExCopyDepth	12.89 (7.33)^1^	0.28 (0.33)^2^	218	24.22
XHMM	4.44 (3.56)^1^	0.32 (0.53)^2^	92	10.22
CONTRA	17.88 (16.56)^1^	0.20 (0.20)^2^	896	99.56

TP/FP ratio for each program was calculated using CNVs identified from 9, 1000 genomes samples run in both computational programs and exaCGH; Average true positive (AvgTP) = TP/9; TP/FP ratio = TP CNV count /FP CNV count; Average CNVs per sample = Total CNV count/9.

^1^Average true positive calculated by excluding CNVs in X and Y Chromosomes.

^2^TP/FP ratio calculated by excluding CNVs in X and Y Chromosomes.

^3^Total number of CNVs predicted by each program excluding CNVs in X and Y Chromosomes (CNV counts for each program including X and Y Chromosomes are presented in Figure 1b).

^4^Average CNVs per sample calculated from counts presented in total CNV count column in Table 2.

False positive (FP) CNVs, defined as CNVs which were predicted only by the computational algorithms, were then identified. Next, these FP CNVs were compared to the exaCGH array design and further analyzed. In order to be conservative in FP identification, only CNV regions that have at least 4 probes in the exaCGH design were selected as the final set of FP CNVs. Then, the ratio between TP and FP CNVs (TP/FP ratio) was calculated for each program (Table 2). CoNIFER (0.92) presented the highest TP/FP ratio indicating the lowest FP count. ExomeDepth (0.67) and ExCopyDepth (0.34) showed second and third highest TP/FP ratio indicating lower FP CNV counts. The lowest TP/FP ratio (with highest FP counts) was exhibited by ExomeCopy and CONTRA (0.20).

Next, we excluded CNV calls from the X and Y chromosomes and the TP/FP ratio was calculated for each program to study the effect of using male and female exomes in a single reference pool. The resulting TP/FP ratios were higher than the previously calculated ratios and indicated lower FP CNV count for autosomal chromosomes. The difference in the TP/FP ratio demonstrated the effect of using male and female samples in the same reference pool. Thus, splitting the reference exome collection based on gender (when large sample collections are available) will improve the CNV calling of X and Y chromosomes.

When considering CONTRA results (Additional file 1: Table S1), it was observed that CONTRA predicts the coordinates of each exon with copy number alteration while all the other programs predict CNVs with one or multiple exons. Thus, in order to find CONTRA CNVs that contain one or multiple exons, adjacent exons were merged and considered as single CNVs, leaving isolated exons as CNVs with single exons. This set of CNVs are referred to as CONTRAmerged. Next, CONTRAmerged CNVs were compared to exaCGH results and TP, AvgTP and FP were identified. The TP CONTRAmerged CNV count was 67 (AvgTP: 7.5). CONTRAmerged CNV counts showed a clear decrease compared to the initial CONTRA results (AvgTP: 17.88). The FP CONTRAmerged count was 826 and the TP/FP ratio was 0.08. This is the lowest TP/FP ratio for all programs, and indicates that CONTRAmerged CNVs exhibits decreased performance in the implemented merge process. Since the next stages of our study are focused on further analysis of CNVs with one or multiple exons, CONTRA results were excluded from downstream analysis and CNVs predictions from other programs (without post-processing CNV calls) were utilized.

### Analysis of CNVs that cover 1–4 exons

In order to evaluate the utility of computational programs in detecting shorter CNVs (1–4 exons), we further analyzed the exon count of each predicted CNV. We counted the number of exons within each predicted CNV, and CNVs containing 1–4 exons were identified. The TP/FP ratio for each program was then calculated (Table 3). CoNIFER, ExomeDepth and XHMM did not identify any TP short CNVs. The intersection of ExomeCopy and ExCopyDepth showed the highest TP/FP ratio, demonstrating the ability of ExomeCopy and ExCopyDepth to detect CNVs that cover 1–4 exons with the highest FP CNV count.

**Table 3:** **True positive (TP), false positive (FP) and TP/FP ratio for short CNVs (1–4 exons)**

Program	1000 genomes exomes			Primary immunodeficiency patients		
	TP	FP	TP/FP ratio	TP	FP	TP/FP ratio
CONIFER	0	0		4	6	0.67
XHMM	0	4		7	32	0.22
ExomeDepth	0	2		50	301	0.17
ExomeCopy	914	6591	0.14	161	1076	0.15
ExCopyDepth	63	267	0.24	52	669	0.08
Intersection of ExomeCopy/ExCopyDepth	34	76	0.45	44	478	0.09

TP, True positive CNVs (CNVs identified both exaCGH and computational programs); FP, False positive CNVs (CNVs identified only by computational programs but not by exaCGH); TP/FP ratio, Ratio between true positive and false positive CNVs.

### Mutation identification using exomes from primary immunodeficiency patients

Since we only tested nine 1000 genome samples with the exaCGH array, and we wanted to further test the utility of computational programs in detecting shorter CNVs, we then ran CNV prediction programs on nine exome samples from patients with primary immunodeficiency disorder (PID). Overlapping CNVs from ExomeCopy and ExCopyDepth were identified by performing an intersection between ExomeCopy, ExCopyDepth results. These results were then mapped to the 1000 genomes exome definition file and CNVs which were covered by 1 to 4 exons were identified.

In parallel to computational CNV prediction, DNA samples from the patients were analyzed by exaCGH and CNV calling was performed using the same criteria applied to the 1000 genomes exaCGH experiments. The TP, FP and TP/FP ratio was calculated for CNVs that cover 1–4 exons for each program. As shown in Table 3, all the programs were able to detect short exonic CNVs. ExomeCopy (161), ExCopyDepth (52) and ExomeDepth (50) presented higher TP CNV counts compared to other two programs. Moreover CoNIFER showed the lowest FP count for detecting shorter CNVs for the PID exome collection. This study of shorter exonic CNVs using primary immunodeficiency patients exhibited the applicability of CoNIFER with lower FP CNVs, and ExomeCopy, ExCopyDepth and ExomeDepth with higher TP CNV counts.

The comparison of short CNV detection showed a higher performance for the intersection of ExomeCopy and ExCopyDepth in 1000 genomes samples compared to the PID group. We reasoned that sequence coverage differences between the two exome groups may contribute to the contrast in short CNV detection. The percentage of bases covered by at least 15 reads in 1000 genomes exomes ranges from 87%-93%, while PID exomes have a very narrow range (98%-99%). This higher variability of depth of coverage across the samples in 1000 genomes collection compared to PID group may contribute to the difference in the performance. In addition, CNV calling of 1000 genomes exomes was performed by splitting the 30 exomes into 3 groups (with 8, 9 and 13 exomes per group) when generating reference collections (Additional file 1: Table S2) whereas PID CNV calling was performed using a reference collection with 22 samples. When considering previous benchmark studies on ExomeDepth, CoNIFER and XHMM, these programs were tested using large reference collections (eg. authors of CoNIFER have tested the program using 366 exomes [5]) and XHMM explicitly claims that it is designed to call CNVs from reference collections with at least 50 exomes [20]. Thus, in addition to the higher variation of read depth across the reference pool, we believe that the use of small sample collections in 1000 genomes exomes also contribute to decrease the performances of ExomeDepth, CoNIFER and XHMM compared to the intersection of ExomeCopy and ExCopyDepth.

**Figure 3:**
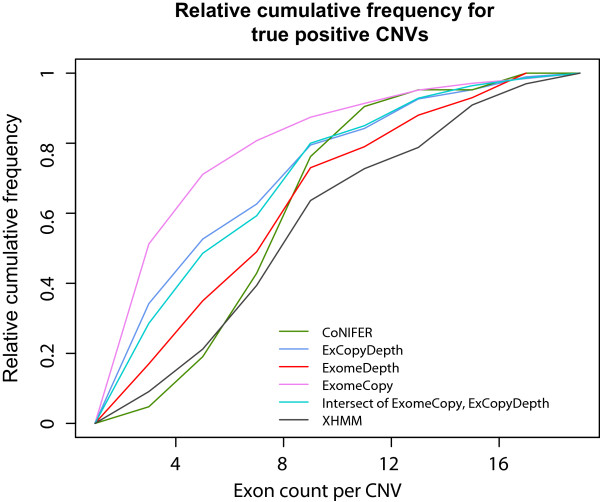
**Relative cumulative frequency for true positive CNVs.** In order to clearly highlight the proportion of short CNVs (with 1–4 exons) predicted by each program, relative cumulative frequency distributions were presented using only the TP CNVs with 0 to 20 exons.

In order to further evaluate short CNV prediction of each program, we used CNVs identified from both exome groups (1000 genomes exomes and PID patients) and examined the number of exons within each predicted TP CNV. Here we compared the relative cumulative frequency distributions of exon count per TP CNVs predicted by computational algorithms. Figure 3 clearly shows that only a small proportion of the CNVs identified by CoNIFER and XHMM contained 1–4 exons compared to ExomeCopy and ExCopyDepth. For example, ~15% of CoNIFER and XHMM TP CNVs contain 1–4 exons, while ~40% of intersection of ExomeCopy and ExCopyDepth and ~45% of ExCopyDepth and ~60% of ExomeCopy identified TP CNVs with 1–4 exons. Analysis of the exon count distribution from each program clearly demonstrated the improved performances of ExomeCopy, ExCopyDepth and the intersection of ExomeCopy and ExCopyDepth in detecting shorter CNVs compared to ExomeDepth, XHMM and CoNIFER. Hence, we further studied the statistical models and methods implemented in each program and detailed comparison of these algorithmic features affecting short CNV prediction is presented in Additional file 1: Text S1.

### Analysis of the copy number state (CNS) predicted by each program

We analyzed TP CNVs to study the consistency of computational algorithms in predicting the CNS of a genomic locus. To do this, we compared the computationally predicted genomic regions from each exome (1000 genomes exomes and PID patient exomes) to the corresponding exaCGH result. Then we studied the consistency of predicted CNS of each locus by intersecting the results of multiple program combinations (Table 4). The results show that ExomeCopy, ExCopyDepth, ExomeDepth and CoNIFER predicted the same CNS only for 50% of the regions detected by all 4 programs. For only 4 regions did all 5 programs agree on the CNS. In contrast, 91.2% of ExCopyDepth and ExomeDepth predictions and 97.4% of ExomeCopy, ExCopyDepth and ExomeDepth predictions showed the same CNSs. The observed 8.8% and 2.6% difference in CNS is due to the difference in the segmentation implemented in ExCopyDepth and ExomeDepth. With the analysis of CNSs, it is important to note that computational algorithms were better at predicting copy number variable regions rather than actual CNSs.

**Table 4:** **Analysis of copy number state (CNS) predicted by different program combinations**

Program combinations	Number of genomic regions with different CNS	Number of genomic regions with same CNS	% of genomic regions with same CNS
ExomeCopy, ExCopyDepth	0	9	100.00
ExomeCopy, ExCopyDepth, ExomeDepth	1	37	97.37
ExomeCopy, ExCopyDepth, ExomeDepth, CoNIFER	3	3	50.00
ExomeCopy, ExCopyDepth, ExomeDepth, CoNIFER, XHMM	0	4	100.00
ExomeCopy, ExCopyDepth, ExomeDepth, XHMM	1	9	90.00
ExomeCopy, ExomeDepth	0	4	100.00
ExCopyDepth, ExomeDepth	3	31	91.18
ExCopyDepth, ExomeDepth, XHMM	0	4	100.00

### Evaluation of the performance of computational CNV prediction algorithms

In order to assess the performance of the programs used, we calculated the sensitivity and false positive rate (FPR) using experimentally validated computational predictions. In addition to TP and FP CNVs, false negative (FN) CNVs were also identified from both exome groups for sensitivity calculations. FN CNVs were considered as the CNVs which were detected only by the exaCGH array experiments but not predicted by computational programs.

**Figure 4:**
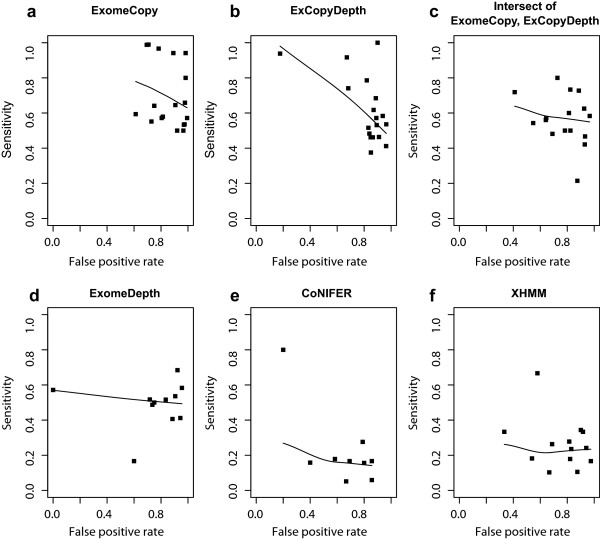
**Sensitivity versus false positive rate for CNV prediction. (a)** ExomeCopy. **(b)** ExCopyDepth. **(c)** Intersection of ExomeCopy and ExCopyDepth (overlapping CNVs predicted by ExomeCopy and ExCopydepth). **(d)** ExomeDepth. **(e)** CoNIFER. **(f)** XHMM. Sensitivity = true positive CNVs/(true positive CNVs + false negative CNVs). False positive rate = false positive CNVs/(false positive CNVs + true positive CNVs).

Next, sensitivity and FPR were calculated for each program (Figure 4). ExomeCopy had higher FPR compared to the other programs while CoNIFER had the lowest false positive rate. When considering the lower sensitivities observed for ExomeDepth, CoNIFER and XHMM, it is important to note that these programs were optimized to detect rare CNVs. CoNIFER and XHMM were further optimized to detect CNVs containing 3 or more exons [15]. Optimizations implemented in these algorithms contribute to the high FN count and affects the sensitivity, as acknowledged in previous studies [4]. When comparing ExomeCopy and ExCopyDepth, sensitivity scores of ExCopyDepth were spread over a wider range (0.4-1). Overlapping CNVs identified from the intersection of ExomeCopy and ExCopyDepth indicated a slight decrease in the sensitivity and a clear decrease in the FP rate compared to ExomeCopy. For example, 7 samples (38.9%) showed very high FPRs (FPR > 0.95) for ExomeCopy (Figure 4a) while only 1 sample (5.5%) showed FPRs over 0.95 for intersection of ExomeCopy and ExCopyDepth (Figure 4c).

With the evaluation of the performance of CNV prediction algorithms, the clinical utility of these algorithms was tested in patients with primary immunodeficiency (PID). The PID patient exomes were studied for disease-related variants, and in one patient we identified homozygosity for a previously reported disease-causing single-nucleotide variant in the Fanconi-associated gene *FANCA* [21]. However, there was no known family consanguinity and compound heterozygosity was expected. Further examination of the region surrounding this variant revealed a low relative read coverage and several succeeding homozygous variants, stretching from *FANCA* exon 26 to 37 (NM_000135.2), suggesting a deletion at this locus. Analysis of CNVs showed that ExomeCopy, ExCopyDepth, ExomeDepth and CoNIFER (all the programs except XHMM) predicted the deletion between exon 26–37 in *FANCA*. exaCGH validated the result, showing a deletion covering the same region in *FANCA* (Additional file 1: Figure S5), and confirmed the higher sensitivity observed for these programs.

In summary, ExomeCopy, ExCopyDepth and the intersection of ExomeCopy showed a higher performance in short CNV prediction compared to ExomeDepth, XHMM and CoNIFER (Figure 3). Moreover, all the computational prediction programs have high FPRs (Figure 4). Thus, in order to predict and to confirm short exonic CNVs, we propose a protocol with two stages combining computer predictions and custom CGH array (exaCGH) experiments. Stage one: initial CNV prediction by selecting the program or program combination depending on the target exome collection and user requirement. Stage two: validation of computational predictions using exaCGH experiments. When selecting a program (or program combination) for short CNV detection, the intersection of ExomeCopy and ExCopyDepth should be used as it demonstrated better performance in detecting shorter CNVs compared to ExomeDepth, XHMM and CoNIFER (Figure 3) with lower FPR compared to ExomeCopy. Importantly, the intersection of ExomeCopy and ExCopyDepth showed improved performance compared to other programs when relatively small sample collections (8–13 exomes) were used for the reference pool or when read depth of the exomes in a reference pool varied over a wide range (TP/FP ratios of 1000 genomes exomes, Table 3). ExomeDepth can be selected to predict rare exonic CNVs with higher sensitivity compared to CoNIFER and XHMM (Figure 4d) when relatively large exome collections were available. However, it is important to note that short CNV detection of ExomeDepth is superior only to CoNIFER and XHMM (Figure 3). If the user requirement is to reach the lowest FPR in predicting CNVs (regardless of the lowest short CNV detection), CoNIFER can be selected (Figure 4e). As discussed earlier, all the programs demonstrated high FPR and showed inconsistency in predicting copy number state (Table 4). Thus, computationally predicted CNVs can be validated using exaCGH experiments (stage two of our protocol) and true positive CNVs can be confirmed.

In an era where many exome sequencing projects are moving toward detecting disease associated variants in lists of candidate genes, our proposed two stage protocol can be implemented to detect and confirm short CNVs. Stage one can be implemented to predict short CNVs in candidate genes, this overcomes the large FP rate when looking genome-wide. Stage two can then be used to validate predicted CNVs by exaCGH.

## Conclusions

In summary, computational methods for CNV identification show clear variation in the number and size of predicted CNVs. Evaluating the sensitivity and false positive rate of computational programs identified the highest sensitivity for ExomeDepth in detecting rare CNVs and the lowest false positive rate for CoNIFER. Following the analysis of short CNVs (1–4 exons), we designed a protocol to identify shorter exonic CNVs by combining computational prediction and custom aCGH methods, which has high relevance for mutation detection in candidate gene studies.

## Electronic supplementary material

Additional file 1: Figure S1: CNV discovery pipeline. **Figure S2.** Flow and implementation of the in-house designed ExCopyDepth algorithm. **Figure S3.** Agilent HD-Probe design and quality control. **Figure S4.** Probe count distribution of exaCGH, Agilent 1x1M array and Affymetrix CytoScan HD. **Figure S5.** Identification and experimentally validation of a CNV in a disease-causing gene using computational predictions and CGH array. **Table S1.** Parameters used for each program. **Table S2.** Summary of the technologies used to derive the exomes used in the study. **Table S3.** 1000 genomes sample IDs of the exomes run in our custom CGH array (exaCGH). **Text S1.** Comparison of the statistical mode and the statistical method of each program affecting the ability to detect short CNVs. (PDF 1 MB)
