# Supplementary material for: Identification of copy number variants from exome sequence data
Source: BMC Genomics. 2014 Aug 7;15(1):661. doi: 10.1186/1471-2164-15-661 (PMC4132917; doi:10.1186/1471-2164-15-661)
Supplement: Supplementary file 1 — Additional file 1: Figure S1: CNV discovery pipeline. Figure S2. Flow and implementation of the in-house designed ExCopyDepth algorithm. Figure S3. Agilent HD-Probe design and quality control. Figure S4. Probe count distribution of exaCGH, Agilent 1x1M array and Affymetrix CytoScan HD. Figure S5. Identification and experimentally validation of a CNV in a disease-causing gene using computational predictions and CGH array. Table S1. Parameters used for each program. Table S2. Summary of the technologies used to derive the exomes used in the study. Table S3. 1000 genomes sample IDs of the exomes run in our custom CGH array (exaCGH). Text S1. Comparison of the statistical mode and the statistical method of each program affecting the ability to detect short CNVs. (PDF 1 MB) [file 12864_2014_6348_MOESM1_ESM.pdf]

Supplementary Figure S1 CNV discovery pipeline.

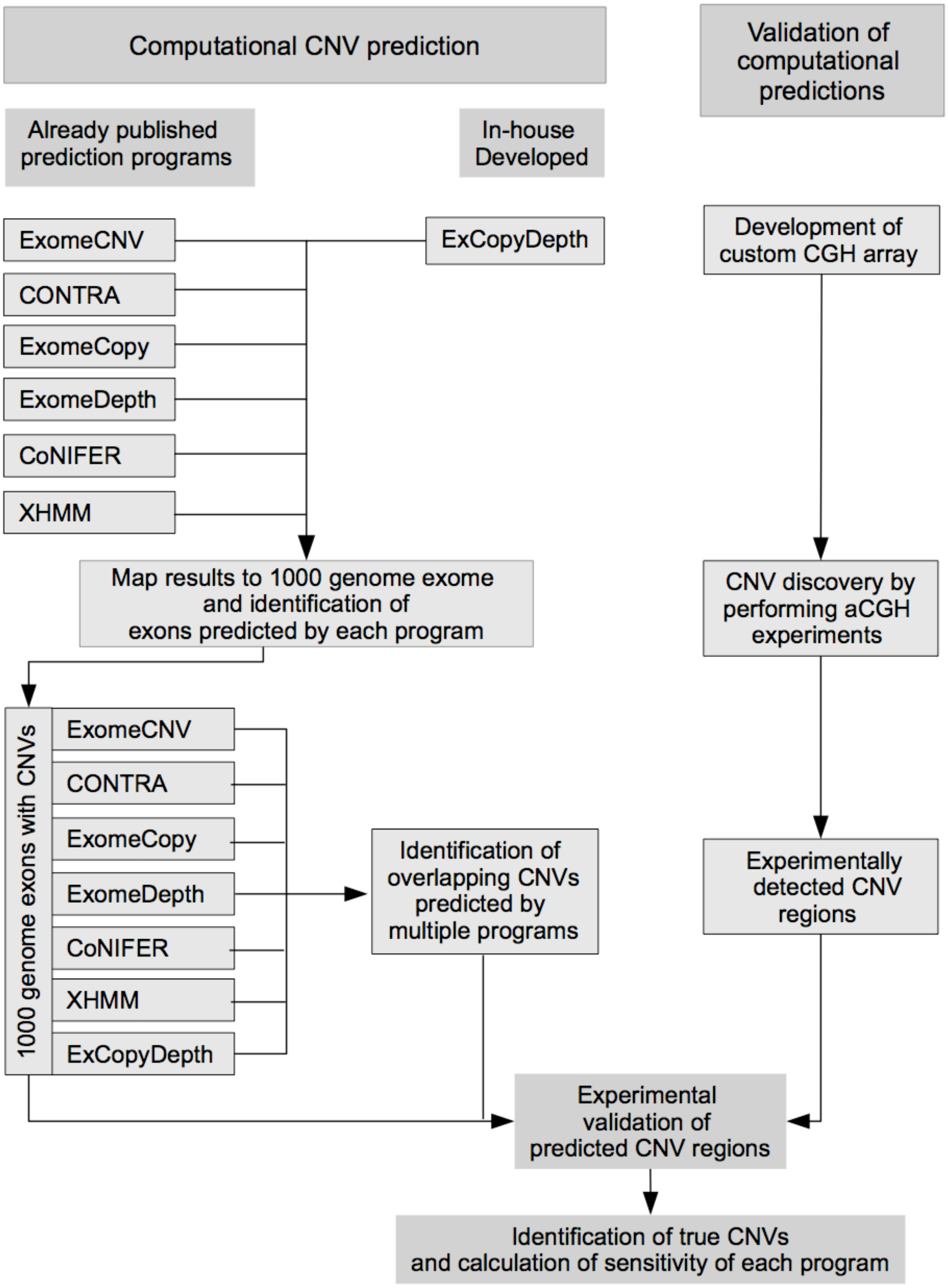

Computational CNV prediction and validation of CNV computationally predicted CNVs using custom CGH array

Supplementary Figure S2 Flow and implementation of the in-house designed ExCopyDepth algorithm.

a.

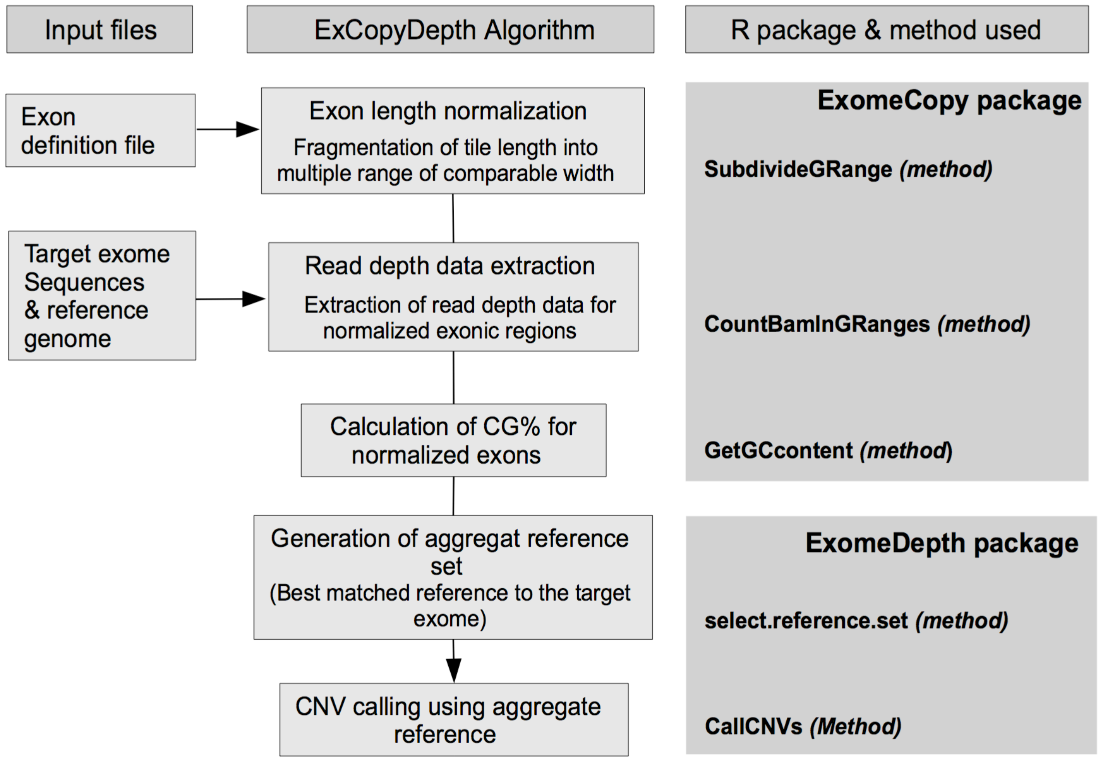

b. Implementation of ExCopyDepth

1. Methods from ExomeCopy package
   a. Load exomeCopy package; **Eg. library(exomeCopy)**
   b. Read exome definition file to a R data frame
   c. Create GRanges object; **Eg. target <- GRanges(seqname=bed.df$seqname,IRanges(start=bed.df$start+1,end=bed.df$end))**
   d. Subdevide the GRange object: Eg. target.sub <- subdivideGRanges(target)
   e. Creating RangedData object; **Eg. rdata <- RangedData(space=seqnames(target.sub),ranges=ranges(target.sub))**
   f. Populate RangedData object with read count data using **countBamInGRanges**: **Eg. rdata[<exome ID>] <- countBamInGRanges(<exome ID >,target.sub)**
   g. Load reference FASTA file: **Eg. target.dnastringset <- scanFa(<reference.fasta>,target.sub)**
   h. Calculate the GC-content;

      Eg. getGCcontent <- function(x) { GC.count <- letterFrequency(x,"GC"); all.count <- letterFrequency(x,"ATGC"); as.vector(ifelse(all.count==0,NA,GC.count/all.count))}
      rdata[["GC"]] <- getGCcontent(target.dnastringset)
   i. Copy read count data of all the samples in a data frame; **Eg. C <- as.data.frame(unlist(values(rdata)[,sample.columns]))**
   j. Calculate back ground read depth, width and GC% squar; **Eg. C.norm <- sweep(C,2,colMeans(C),"/") ; rdata[["bg"]] <- apply(C.norm,1,median); rdata[["width"]] <- width(rdata); rdata[["GC.sq"]] <- rdata$GC^2**
2. Load ExomeDepth package; Eg. library(ExomeDepth)

a. Convert RangeData object to data frame; **Eg. ExomeCount.dafr <- as(rdata[, colnames(rdata)], 'data.frame')**

b. Format the data frame if necessary; **Eg. ExomeCount.dafr$chromosome <- gsub(as.character(ExomeCount.dafr$space),pattern='chr',replacement='')**

c. List column names of the dataframe; **Eg. exomeSamples <- grep("EXOME_SAMPLE+",colnames(ExomeCount.dafr),value=TRUE)**

3. CNV calling ExomeDepth CNV calling steps

a. Select the test exome; **Eg. my.test <- ExomeCount.dafr[[i]] # i is the index number of the column in the dataframe**

b. Select and generate reference exome matrix; **Eg. my.ref.samples <- subset(exomeSamples, exomeSamples != i); my.reference.set <- as.matrix(ExomeCount.dafr[, my.ref.samples]); my.choice <- select.reference.set (test.counts = my.test,reference.counts = my.reference.set,bin.length = (ExomeCount.dafr$end - ExomeCount.dafr$start)/1000,n.bins.reduced = 10000); my.reference.selected <- apply(X = as.matrix(ExomeCount.dafr[, my.choice$reference.choice]),MAR = 1, FUN = sum)**

c. CNV calling using the generated reference set; **Eg.all.exons <- new('ExomeDepth',test = my.test,reference = my.reference.selected,formula = 'cbind(test, reference) ~ 1'); all.exons <- CallCNVs(x = all.exons, transition.probability = 10^-4, chromosome = ExomeCount.dafr$space, start = ExomeCount.dafr$start, end = ExomeCount.dafr$end, name = ExomeCount.dafr$start)**

c. Implementation of ExCopyDepth and ExomeCopy without rerunning tile length normalization

As ExomeCopy and ExCopyDepth use the same tile length normalization method, this step can be executed only once when implementing both ExomeCopy and ExCopyDepth.

Steps to follow when running a single tile length normalization method for both ExomeCopy and ExCopyDepth.

1. Follow step 1 of Supplementary Figure S2b

a. **Run R codes listed in Supplementary Figure S2b step 1 (Methods from ExomeCopy package)**

2. Before moving to step 2 of Supplementary Figure S2b, save the current workspace

a. **Eg. save.image("tileLengthNormalized.RData")**

3. Continue the rest of the steps in ExomeCopy and complete the ExomeCopy run.

4. In order to run ExCopyDepth using the normalized read count data generated earlier, load workspace image created in step 2 of Supplementary Figure S2c.

a. **Eg. load("tileLengthNormalized.RData")**

5. Delete all the objects except the R object that carry read count data (rdata)

a. **Eg. l <- ls(); l <- subset(l, l != "rdata"); rm(list=l); rm(l)**

6. Continue with step 2 and step 3 of Supplementary Figure S2b and complete the ExcopyDepth run.

Supplementary Figure S3 Agilent HD-Probe designing and quality control

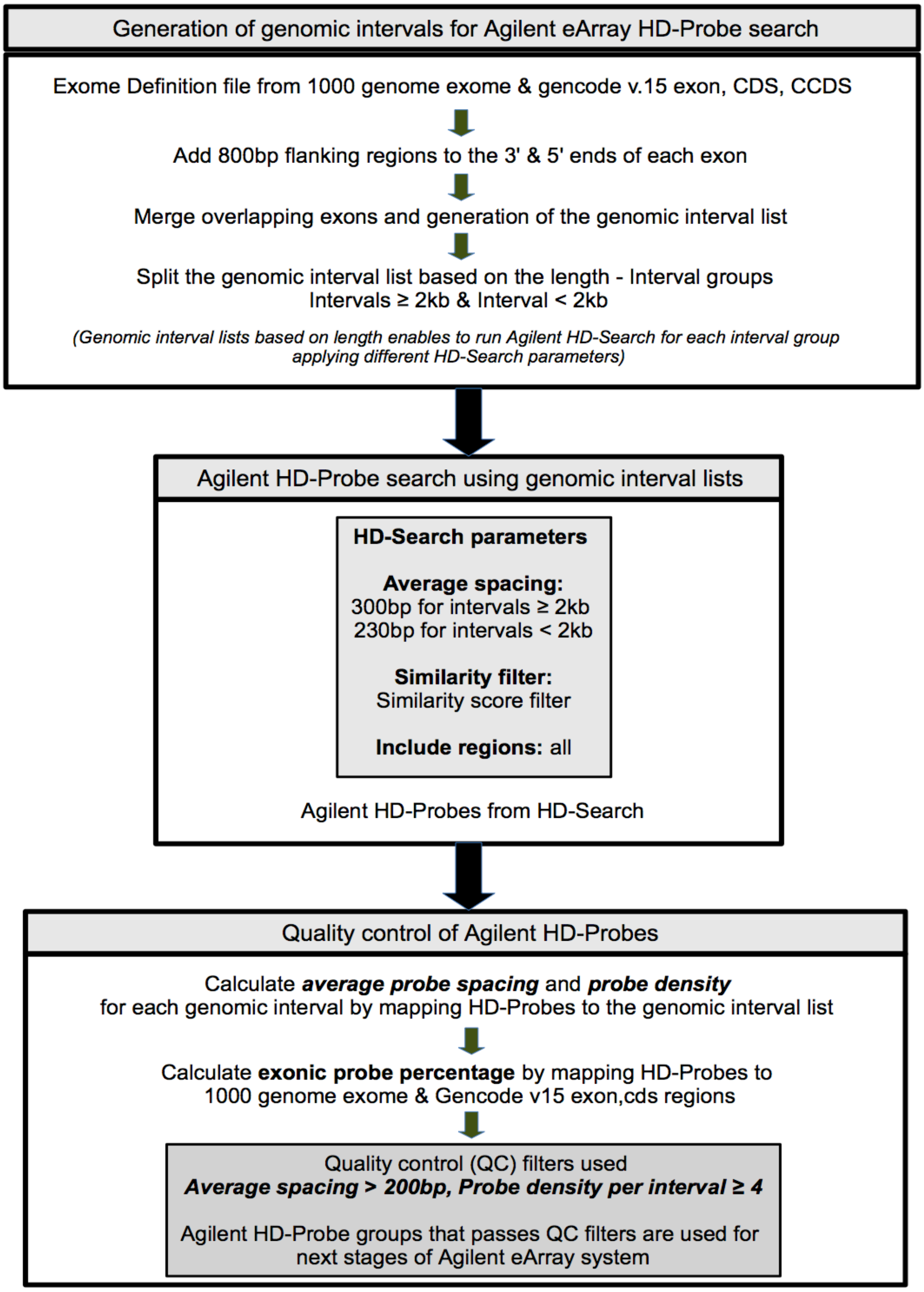

Agilent eArray HD-Probe (High Definition Probe) search is initiated by using a genomic interval list which is generated after adding 800bp flanking regions to the 5’ and 3’ ends of the exons. Following the HD-Probe search resulted probe list was evaluated by using an in-house designed quality control pipeline

Supplementary Figure S4 Probe count distribution of exaCGH, Agilent 1x1M array and Affymetrix CytoScan HD

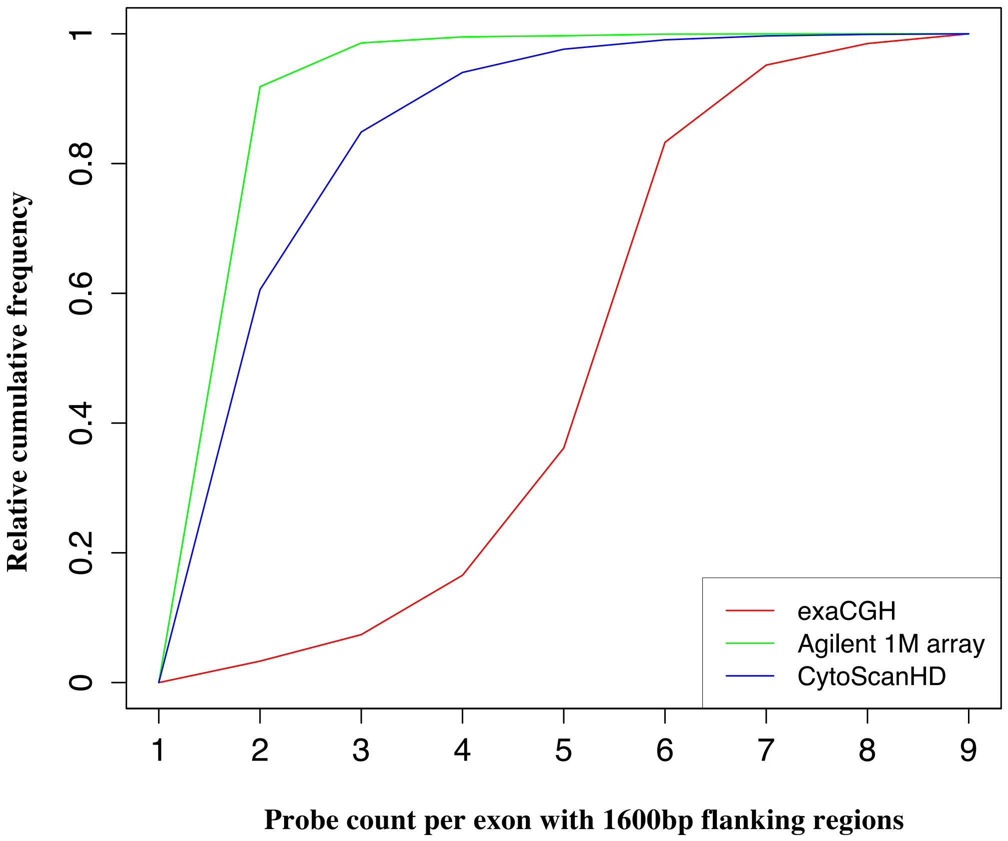

Exonic and CDS regions of GENCODE V.15 including 800bp flanking regions were used as target regions and then number of probes mapped to each target region was calculated when comparing probe coverage of each design.

Supplementary Figure S5 Identification and experimentally validation of a CNV in a disease-causing gene using computational predictions and CGH array

a

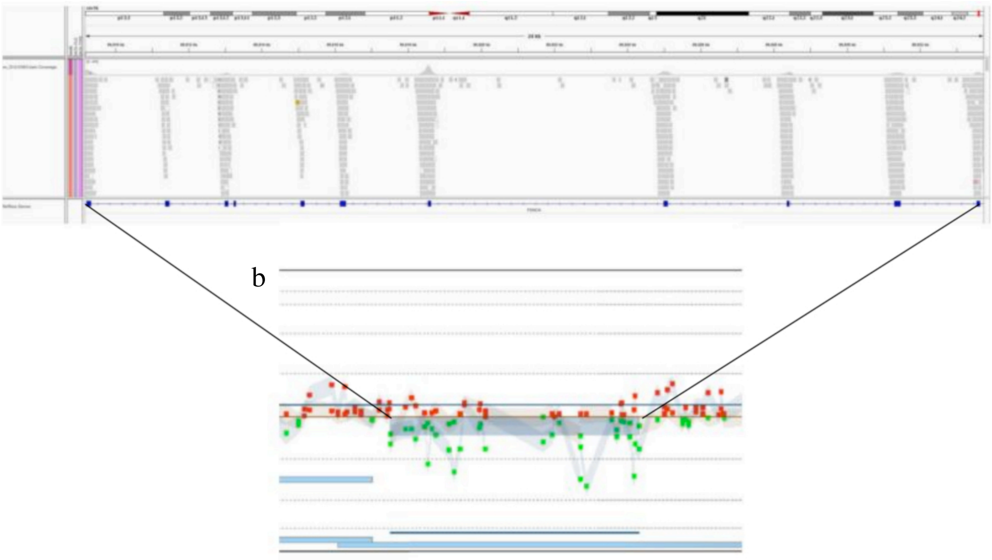

(a) CNV region predicted by computational prediction programs (ExomeCopy, ExCopyDepth, ExomeDepth and CoNIFER). (b) CNV resulted from the exaCGH for the region predicted by computational programs.

Supplementary Table S1 Parameters used for each program.

Program	Parameters	Remarks
ExomeCNV	Thresh-old sensitivity & specificity = 0.9999<br>Optimization Strategy = Specificity<br>Combine exonic CNV into segments<br>Thresh-old sensitivity & specificity = 0.999<br>Optimization Strategy = Averaging between Specificity & Sensitivity<br>Sample admixture rate = 0	CNV detection using ExomeCNV requires a control and case exomes as the input. Therefore an additional 1000 genome exome (**NA12282**) is selected randomly as control. Target exomes that are sequenced and captured using the same method are used as case exomes.
CONTRA	Number of bins = 20 (numBin 20)<br>Exclude all the bases where read depth is less than 10 (minReadDepth)<br>Exclude target regions that are shorter than 10bp in length. (minNBases)	Separate control dataset (baseline sequence) for each target exome group was generated and target exomes were run using the baseline sequence of the respective group. CNVs reported in variant call format (VCF file) were used as the final set of CNV calls from the program
ExomeCopy	subdivided to nearly subsize = 100 (subsize)<br>relative tolerance for convergence used in the optim function = 1e-04, (reltol)<br>possible copy numbers for the different states = 0:4, (S)<br>expected copy number for the normal state = 2, (d)<br>initial setting for probability to transfer to a CNV state = 1e-04, (goto.cnv)<br>initial setting for probability to transfer to a normal state = 1/20, (goto.normal)<br>initialize phi = "norm" (init.phi)	Tiled regions are subdivided to reduced the effect of GC content of the tile and CNV calling is performed by minimizing the bias introduced by the GC%.
ExomeDepth	minimum mapping quality to include a read = 20 (min.mapq)<br>maximum distance between the side of the target region and the middle of the paired read to include the paired read into that region = 300 (read.width)<br>Transition probability = 1e-04 (transition.probability)	Most appropriate reference set for the target exome sequnce is created and CNV calling is performed.

Combined ExomeCoy. ExomeDepth	Same parameters specified in ExomeCopy and ExomeDepth are used here.	Tiled regions are subdivided to reduced the effect of GC content using the ExomeCopy and CNV calling is performed using Most appropriate reference set by the ExomeDepth.
CoNIFER	Number of removed singular values = 6 (k)<br>Threshold for calling = CNVs 1.5 (minimum SVD-ZRPKM)	Multiple number of initial analysis is performed using range of --svd values (between 5 and 12), and svd =6 was selected after examining the scree plot.
XHMM	Exome-wide CNV rate = 1e-08<br>Mean number of targets in CNV = 6 (geometric distribution)<br>Mean distance between targets within CNV = 70 KB (exponential decay)<br>DEL read depth distribution ~ N(mean=-3, var=1)<br>DIP read depth distribution ~ N(mean=0, var=1)<br>DUP read depth distribution ~ N(mean=3, var=1)	XHMM utilizes the GATK DepthOfCoverage,<br><br>GCContentByInterval walkers (McKenna et al., 2010) and the PLINK/Seq program.

Supplementary Table S2 Summary of the technologies used to derive the exomes used in the study

Sequencing center	Sequencing platform	Capture technology	Number of exoms per group
Broad Institute (BI)	Illumina HiSeq 2000	Agilent SureSelect_All_Exon_V2	13
Beijing Genomics Institute (BGI)	Illumina HiSeq 2000	NimbleGen v1 2.1M_Human_Exome	8
Washington University Genome Science Center (WUGSC)	Illumina Genome Analyzer II	Agilent SureSelect_All_Exon_V2	9

Supplementary Table S3 1000 genomes sample IDs of the exomes run in our custom CGH array (exaCGH).

1000 genomes sample ID	NA06986, NA06989, NA06994, NA07037, NA07051, NA07347, NA11843, NA12249, NA12340

Supplementary Text S1 Comparison of the statistical mode and the statistical method of each program affecting the ability to detect short CNVs

ExomeCopy and ExomeDepth use a similar approach in detecting CNVs (read count normalization followed by the Hidden Markov Model (HMM) based CNV calling). However, ExomeCopy differs from ExomeDepth by implementing tile length normalization (segmentation of target regions into regions with nearly equal length) prior to read count normalization. Implementation of tile length normalization of ExomeCopy is the main contributor for the improved performance in detecting short CNVs as claimed by ExomeCopy authors and confirmed by our study. In addition to the generation of an aggregate reference set, ExomeDepth implements a beta-binormial model that accounts for the variability of observed variance of read depth depending on the total read count of each sample. The combination of an improved statistical model and the use of an aggregate reference set in ExomeDepth contributes to the overall decrease in CNV count (compared to ExomeCopy CNV calls), including false positive CNVs (Table 2 and Table 3) and short exonic CNVs. However, as shown in Figure 3, ExCopyDepth (implementation of tile length normalization to ExomeDepth CNV calling) clearly increased the prediction of short exonic CNVs compared to ExomeDepth and confirms the direct effect of tile length normalization in detecting short CNVs. Both CoNIFER and XHMM authors claim the capability of detecting CNVs containing 3 or more exons and implement singular value decomposition (SVD) based method to remove experimental and systematic artifacts. Following the SVD normalization, CoNIFER deviated from XHMM by calling CNVs using hard thresholds on consecutive runs of 3 or more targets with normalized SVD values. Thus CoNIFER shows limitations in predicting CNVs with 1-3 exons. XHMM implements HMM to assess the quality of each CNV signal rather than using hard filters as in CoNIFER. Thus XHMM shows better performance in detecting short CNVs compared to CoNIFER.
